# Moving Multiunit Housing Providers Toward Adoption of Smoke-Free Policies

**Published:** 2010-12-15

**Authors:** Barbara Pizacani, Diane Laughter, Kylie Menagh, Michael Stark, Linda Drach, Colleen Hermann-Franzen

**Affiliations:** Multnomah County Health Department and Oregon Public Health Division; Health In Sight LLC, Portland, Oregon; Oregon Public Health Division, Portland, Oregon; Multnomah County Health Department and Oregon Public Health Division, Portland, Oregon; Multnomah County Health Department and Oregon Public Health Division, Portland, Oregon; American Lung Association in Oregon, Portland, Oregon

## Abstract

**Background:**

Tenants in multiunit housing are at elevated risk for exposure to secondhand smoke at home because of smoke migration from other units.

**Community Context:**

In 2004, tobacco control advocates in the Portland, Oregon, metropolitan area began to address this issue by launching a campaign to work with landlord and tenant advocates, private- and public-sector property managers, and other housing stakeholders to encourage smoke-free policies in multiunit housing.

**Methods:**

We outline the 6-year campaign that moved local housing providers toward adopting no-smoking policies. We used the stages of change model, which matches potential messages or interventions to a smoker's readiness to quit smoking.

**Outcome:**

The campaign resulted in Oregon's largest private property management company and its largest public housing authority adopting no-smoking policies for their properties and a 29% increase in the availability of smoke-free rental units in the Portland-Vancouver metro area from 2006 through 2009.

**Interpretation:**

We learned the importance of building partnerships with public and private stakeholders, collecting local data to shape educational messages, and emphasizing to landlords the business case, not the public health rationale, for smoke-free housing.

## Background

Despite declines in exposure to secondhand smoke among adults in the United States because of state workplace smoking laws, renters in multiunit housing remain at elevated risk for home exposure to secondhand smoke (1). Tenants in multiunit housing can be exposed to secondhand smoke from seepage through walls, wiring, plumbing, and ventilation systems and under doors (2,3). They also can be exposed in common areas or from outside balconies or patios where smoking is allowed.

Many renters and landlords support smoke-free housing. Studies as early as 2001 in Minnesota showed that approximately 55% of renters would be "very likely" to choose a smoke-free building over a smoking-permitted building if other amenities were equal, and those property managers who had adopted no-smoking policies reported being very likely to continue doing so (4). Despite these preferences, many property managers remained reluctant to implement no-smoking policies, citing economic and legal issues (1).

## Community Context

Since 1997, Oregon has had considerable success reducing adult and youth smoking prevalence and protecting almost all workers from secondhand smoke by implementing a smoke-free workplace law that includes restaurants and bars and increasing the proportion of households that prohibit smoking in the home (5). Perhaps because of increased public awareness of the harms of secondhand smoke generated by these public policies, state- and county-level public health workers began to receive requests for help from individual renters about secondhand smoke drifting into their apartments. In 2004, the tobacco control community began to address this issue by launching a campaign to work with landlord and tenant advocates, private- and public-sector property managers, and other housing stakeholders to encourage smoke-free policies in multiunit housing.

This case study outlines lessons learned from the 6-year campaign of the Portland-Vancouver Metro Area Smokefree Housing Project. For purposes of this study, "we" refers to the project leadership team, comprising staff (D.L., K.M., C.H-F., and others) from the initial 3 partner agencies: American Lung Association in Oregon (ALAO); Multnomah County Health Department (MCHD) in Portland, Oregon; and Clark County Public Health (CCPH) in Vancouver, Washington. The remaining authors (B.P., L.D., and M.S.) helped write this case study and conducted a related evaluation of the implementation of a smoke-free policy in housing managed by 1 of the stakeholders (6).

## Methods

### Preparing for the campaign

**Figure 1:**
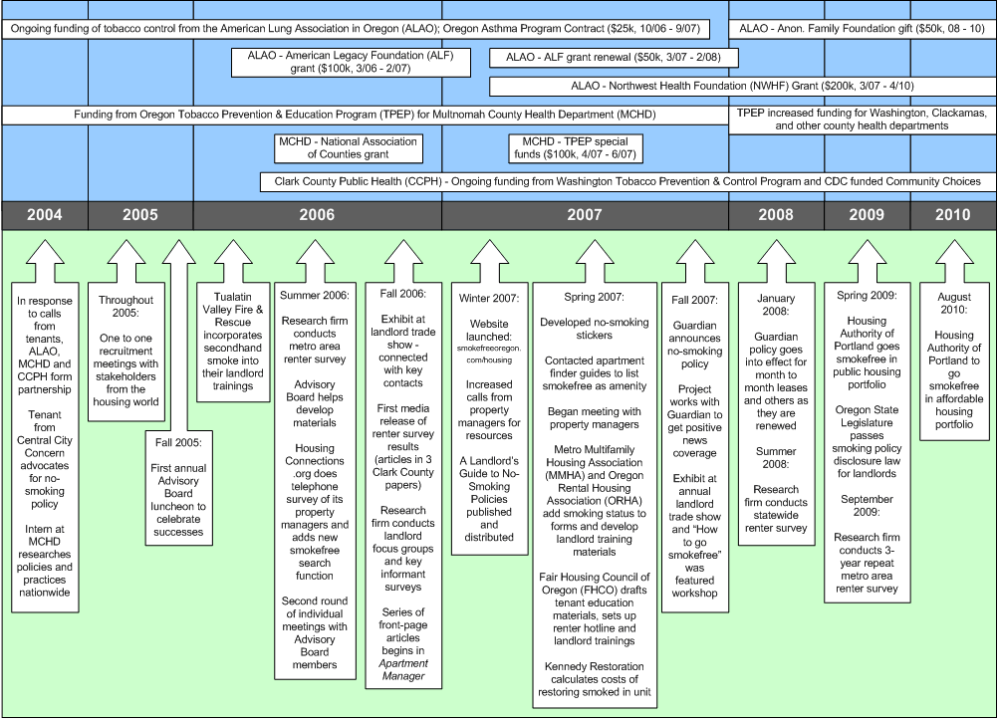
Timeline and funding streams for the Portland-Vancouver Metro Area Smokefree Housing Project.

Before approaching local landlords to introduce the idea of smoke-free housing, we assembled staff for the campaign and gathered information from national colleagues. In 2004, ALAO and MCHD partnered to address secondhand smoke exposure in multiunit housing. In early 2006, CCPH joined the effort, ALAO received start-up funding from the American Legacy Foundation, and the Portland-Vancouver Metro Area Smokefree Housing Project was created. From 2004 to 2006, each organization dedicated approximately one-half of 1 employee's time in in-kind staff time; in 2007, ALAO received a grant from the Northwest Health Foundation that funded another full-time employee for 3 years. In addition to ongoing tobacco use prevention funding at Multnomah and Clark counties, grant funding through April 2010 totaled $530,000 for the project (Figure 1). Project staff gathered information from the Smoke-Free Environments Law Project in Michigan, Association for Nonsmokers — Minnesota, and the Smokefree Housing Coalition of Maine, and honed strategies by talking with colleagues at national meetings and through a national listserve about smoke-free housing.

**Table:** 

This figure provides a timeline of key events and receipt of funds for the Portland-Vancouver Metro Area Smokefree Housing Project. The time covered is from the project’s inception in 2004 through 2010.
Beginning in 2004, the project received funding of tobacco control from the American Lung Association in Oregon (ALAO); funding of $25,000 from October 2006 through September 2007 was through Oregon Asthma Program Contract. Also beginning in 2004, funding came from the Oregon Tobacco Prevention and Education Program (TPEP) for Multnomah County Health Department (MCHD). In 2008, that funding was increased for Washington, Clackamas, and other county health departments.
From March 2006 through February 2007, the project received a $100,000 grant from an American Legacy Foundation (through ALAO); that grant was renewed for March 2007 through February 2008 for $50,000.
Beginning in 2006 and continuing through 2010, Clark County Public Health (CCPH) in Vancouver, Washington, provided ongoing funding from the Washington Tobacco Prevention and Control Program and CDC-funded Community Choices.
In 2006, MCHD received a grant from the National Association of Counties.
From March 2007 through April 2010, the project received a $200,000 grant from the Northwest Health Foundation (through ALAO).
In April through June 2007, MCHD received $100,000 in special funds from TPEP.
From 2008 through 2010, ALAO received an Anonymous Family Foundation gift of $50,000.
Key events on the project timeline were the following:
**2004**
In response to calls from tenants, ALAO, MCHD, and CCPH form a partnership. A tenant from Central City Concern advocates for a no-smoking policy. An intern at MCHD researches policies and practices nationwide.
**2005**
One-to-one recruitment meetings with stakeholders from the housing world are held throughout 2005. In the fall of 2005, the first annual advisory board luncheon is held to celebrate successes.
**2006**
Tualatin Valley Fire and Rescue incorporates secondhand smoke issues into their landlord trainings. In the summer, a research firm conducts a metro area renter survey, the advisory board helps develop materials, HousingConnections.org does a telephone survey of its property managers and adds a new smoke-free search function, and a second round of individual meetings with advisory board members occurs. In the fall, an exhibit at a landlord trade show provides connections with key contacts, the first media release of renter survey results (articles in 3 Clark County papers) occurs, a research firm conducts landlord focus groups and key informant surveys, and a series of front-page articles begins in Apartment Manager.
**2007**
In the winter, smokefreehousinginfo.com website is launched, there are increased calls from property managers for resources, and *A Landlord’s Guide to No-Smoking Policies* is published and distributed. In the spring, the project developed no-smoking stickers, contacted apartment finder guides to list smoke-free as an amenity, began meeting with property managers, the Metro Multifamily Housing Association (MMHA) and Oregon Rental Housing Association (ORHA) add smoking status to forms and develop landlord training materials, the Fair Housing Council of Oregon (FHCO) drafts tenant education materials and sets up renter hotline and landlord trainings, and Kennedy Restoration calculates the costs of restoring a smoked-in unit. In the fall, Guardian announces its no-smoking policy, the project works with Guardian to get positive news coverage, and an exhibit at an annual landlord trade show and “How to Go Smokefree” was a featured workshop.
**2008**
In January, the Guardian policy goes into effect for month-to-month leases and others as they are renewed. In the summer, a research firm conducts a statewide renter survey.
**2009**
In the spring, the Housing Authority of Portland goes smoke-free in its public housing portfolio, and the Oregon State Legislature passes smoking policy disclosure law for landlords. In September, a research firm conducts 3-year repeat metro area renter survey.
**2010**
In August, the Housing Authority of Portland is to go smoke-free in its affordable housing portfolio.

It became clear that these strategies needed to be matched to stakeholders' level of readiness for policy change, since few landlords or managers were considering no-smoking policies in 2004. The concept of stage-matching an intervention or strategy is familiar to many in public health and tobacco control because the stages of change model (7) encourages matching potential messages or interventions to a smoker's readiness to quit. We learned that continually making the business case for smoke-free housing was an effective motivational strategy for moving stakeholders through the early stages of change (precontemplation, contemplation, and preparation) into making and maintaining the change (action and maintenance).

### Precontemplation to contemplation: developing an advisory board and conducting formative evaluation

In the stages of change model, precontemplation refers to those who are not yet considering behavior change, and contemplation refers to those who are considering change but not immediately. As noted, smoke-free policies were not an industry norm in 2004, and we knew from national data (1) that many housing providers were concerned about whether such policies would result in lost revenue or cause legal problems. We decided, therefore, to conduct informational interviews with stakeholders over coffee or lunch, using a low-key, collegial approach to address concerns and stimulate interest. Stakeholders included members of landlord trade associations, renter advocacy groups, the Fair Housing Council of Oregon, local housing authorities, and agencies that provide training and communication to property managers (eg, fire and police departments). We asked each if they would be interested in participating as advisory board members. All of the people who were identified as potential board members agreed, and many helped us recruit other key partners. The advisory board's initial focus was to guide the development and implementation of several assessment activities designed to measure the scope of renters' exposure to secondhand smoke, market demand for smoke-free living, and property managers' opinions about no-smoking policies. Advisory board responsibilities are listed in the Box, and the Appendix lists the organizations represented on the board.

**Box. Major Tasks of the Advisory Board**

- Identify other stakeholders for advisory board membership and for other campaign activities
- Define local data needs; assist with instrument development and data collection
- Develop the business case for smoke-free housing
- Create educational materials and tools for property managers and tenants
- Identify venues for disseminating information
- Champion the cause of smoke-free multiunit housing in their organizations and the community

In the summer of 2006, we contracted with an independent research firm to conduct a random-digit–dialed, population-based survey of 356 metro-area renters; 26% were current smokers. Survey results revealed 3 key findings: most renters who smoke already smoke outside, 75% of renters in the Portland-Vancouver metro area would rather live in nonsmoking buildings, and 52% of renters said they would even pay a little extra rent for smoke-free housing (8). This last finding does not mean that smoke-free units will or should cost more, but rather signals to landlords that renters view smoke-free as a desired amenity and that going smoke-free could help attract qualified applicants. A follow-up statewide survey in 2008 (n = 300, 33% were smokers) had similar findings, showing that approximately two-thirds of renters in the rest of the state preferred smoke-free housing and approximately 40% would pay more for it (9). These data showed our landlord partners that no-smoking policies were a business opportunity, a message they began to share within their organizations and trade associations.

The same research firm also conducted 6 landlord focus groups, with a total of 32 participants (9), and 10 key informant interviews with property managers and opinion leaders in the summer of 2006 (10). These qualitative data helped us understand why property managers would or would not adopt no-smoking rules and helped define practical issues related to policy adoption, including potential implementation strategies. They also allowed us to test several messages that were based on national examples and local renter survey data. We found that local property managers already knew that allowing smoking was costing them money and that it increased fire risk, but they worried about whether no-smoking policies were legal and whether there was sufficient tenant demand for units covered by such a policy. Property managers also said they lacked the tools needed for policy implementation (eg, appropriate lease language). Other findings were that tenant health was not a sufficient motivator for them to change their policies and that they trusted their own trade associations for information and guidance, not public health agencies. Finally, they showed a strong preference for educational materials with a business look and language.

### Contemplation to preparation: developing educational materials and communicating the message

The next step was to present the focus group and survey data to the advisory board. Using this information, the board helped produce informational pieces for landlords and renters such as the printed guide *A Landlord's Guide to No-Smoking Policies* (11) (www.smokefreehousingnw.com/landlords/Landlord's%20Guide%20to%20No-Smoking%20Policies%20third%20version%204-10.pdf) and a website (12). Board members provided practical tools for policy implementation, such as lease language, signage, and tenant notification letters. The website features tools for both landlords and renters and had more than 11,000 home page hits from July 2007 through December 2009.

Involving key leaders from the housing sector as advisors throughout the assessment process resulted in credible data that addressed the specific concerns of decision makers and opinion leaders. This involvement also ensured that advisory board members were invested in the findings and willing to communicate key messages through their websites, newsletters, and training programs.

Our next step was to develop a communication strategy that could answer questions and provide tools. Our strategy was built on existing relationships and resources such as housing sector trade shows, trainings, industry publications and listserves, and business publications, and print and broadcast media for general audiences. Successes in the fall of 2006 included staffing a display at the major property manager trade show in our area and soliciting a commitment from the trade newspaper *The Apartment Manager* to run a monthly series of front-page articles on smoke-free housing that continued for 26 months. In 2007 and 2008, we facilitated a conference workshop on adopting no-smoking policies and worked with mainstream media to develop stories about the smoke-free project. After each trade show, we contacted the largest or most influential property management companies that were present, scheduled meetings with managers, and provided them with tools to implement no-smoking policies.

The City of Portland and the Fair Housing Council of Oregon were key partners in developing resources for tenants. We augmented the city's free housing-finder website (HousingConnections.org) instead of creating a stand-alone registry that would require staff time to maintain. A grant from the National Association of Counties funded a new feature to sort properties by smoking status and a landlord survey to ensure that smoking policies were described accurately for the listings.

The Fair Housing Council of Oregon continued to provide outreach and education to landlords through existing landlord training programs and developed several resources for renters, including educational materials on when and how to pursue protection from secondhand smoke under fair housing laws. In addition, the council staffed a renter hotline to help tenants seek reasonable accommodation if they had a pre-existing chronic health condition or other disability aggravated by secondhand smoke. Finally, we urged apartment guides and online listing services to include "no-smoking" as a featured amenity, and 4 have done so (Housing Connections, Apartment Guide [web and print versions], OregonLive.com, and RentLinx). Additionally, the websites ShowMeTheRent.com (which uses RentLinx information) and Rentals.com also offer smoke-free in their housing search criteria.

## Outcome

### Action: policy changes

Two local housing providers — Guardian Management LLC and the Housing Authority of Portland (HAP) — adopted no-smoking policies. In January 2008, Guardian Management LLC, Oregon's largest property management company with 6,500 units in Oregon and 1,500 in 6 other states, prohibited smoking on the properties they own or manage, which include high-end, market-rate units and subsidized, low-income housing. When some tenants complained to the media about the policy, we helped Guardian create positive media messages, resulting in favorable coverage. Guardian sent out a notification letter approximately 3 months before the policy went into effect on January 1, 2008, for most tenants on month-to-month leases; the few tenants with annual leases were required to comply as their leases were renewed over the following months. In an evaluation of a group of 17 rent-subsidized Guardian buildings, most tenants reported that the smoke-free policy was acceptable in a survey conducted 5 months after implementation (6). Over a 1-year follow-up in this group of buildings, only 6 Guardian residents reportedly left because of the policy, and 1 of those later tried to return (B. Pizacani, March 2009). Guardian made its notification letter, no-smoking lease addendum, warning letter, and changes in House Rules for Rural Development Properties available to other property managers (12).

In August 2009, HAP prohibited smoking in all of its 1,993 public housing units, and in August 2010 it instituted the same policy in its 3,760 affordable housing units. Although stakeholders from both HAP and Guardian found the business case for smoke-free policies compelling, those from HAP additionally cited health and safety of residents and site staff as a reason for the policy change. HAP requested additional support and information regarding health education for dangers of secondhand smoke and links to cessation services. Both Guardian and HAP participated in workshops for their onsite managers, some of whom smoke, to help them deal with enforcement challenges, feel more comfortable talking to residents about their smoking, and refer tenants who want to quit smoking to appropriate resources. Details about the steps taken by HAP, the implementation tools it developed (13), and its enforcement plan (14), which includes referral to smoking cessation resources, are available on the Internet.

In July 2009, ALAO and project partners surveyed 300 metro-area renters (23% of whom were current smokers) to assess changes in no-smoking policies since the 2006 survey. Findings showed a 29% increase in the availability of smoke-free rental units in the Portland-Vancouver metro area, amounting to approximately 13,000 new smoke-free units in the 3-year period (15).

The 2009 Oregon legislature passed a law that requires Oregon landlords, as they sign residential rental agreements, to disclose their smoking policy to their tenants. According to the law, "The disclosure must state whether smoking is prohibited on the premises, allowed on the entire premises or allowed in limited areas on the premises. If the smoking policy allows smoking in limited areas on the premises, the disclosure must identify the areas on the premises where smoking is allowed" (16). The law went into effect January 1, 2010. Key to the bipartisan success of the bill was the fact that the landlord trade associations and fair housing advocates, both crucial partners in the project from the beginning, supported the bill.

### Maintenance: smoke-free as a social norm

After adopting a no-smoking policy in January 2008, a high-level manager at Guardian and past president of an influential landlord trade association became a champion of smoke-free housing locally, regionally, and even nationally. Guardian's policy changes and the champion's support have been invaluable, as property managers tend to emulate large and successful property management companies.

Our project resulted in sustainable systems-level changes that are setting a new industry standard and facilitating the change to smoke-free policies for other landlords. Examples of these changes include the following:

- Incorporation of messages about the benefits of smoke-free housing into major sources of landlord information, including trainings, newsletters, websites, and trade shows.
- Incorporation of smoking status into lease forms by the 3 main providers in the region.
- Landlord requirements to disclose their smoking policy as part of their rental agreements.

**Figure 2:**
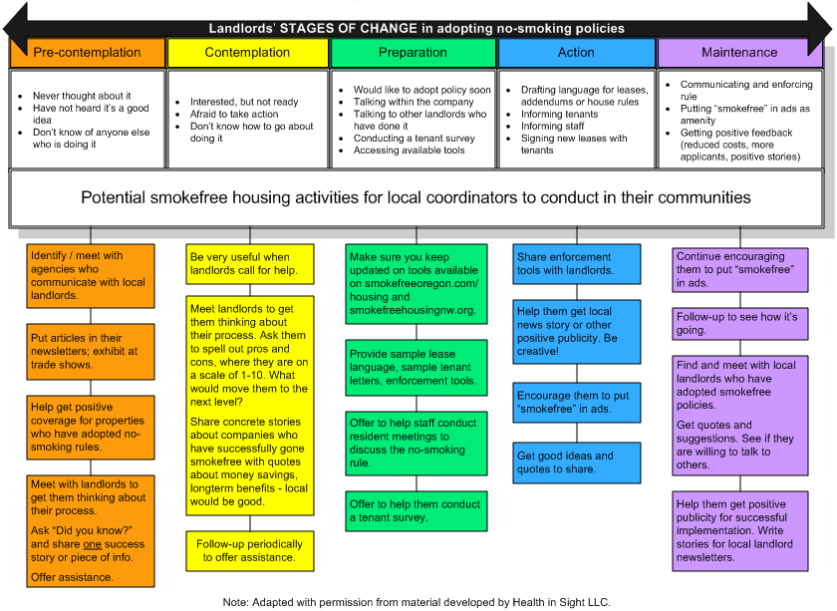
Potential activities to move landlords toward implementing and maintaining smoke-free housing, Oregon Smokefree Housing Project.

In addition, our model was adopted by the Oregon Tobacco Prevention and Education Program (TPEP), which initiated a new contract with Health In Sight LLC for coordinating smoke-free housing efforts at the state level. TPEP also requires grantees in all 36 counties in Oregon to work with property managers to adopt no-smoking policies. A schematic outlining activities to accomplish this, depicted in terms of a landlord's stage of change, was developed as a tool for these county programs (Figure 2).

**Table:** 

This figure presents activities that can be conducted to move landlords along the stages of change toward implementing and maintaining smoke-free housing. The 5 stages of change are precontemplation, contemplation, preparation, action, and maintenance. Each stage of change is described along with suggested activities for that stage.
**Precontemplation**
In this stage, landlords may have never thought about smoke-free housing, have not heard that smoke-free housing is a good idea, and do not know of anyone else who is doing it. Potential smoke-free housing activities for local coordinators to conduct in their communities with landlords in the precontemplation stage were the following:
Identify and meet with agencies who communicate with local landlords. Put articles in landlord trade newsletters; exhibit at trade shows. Help get positive news coverage for properties who have adopted no-smoking rules. Meet with landlords to get them thinking about their process. Ask: “Did you know?” and share one success story or piece of information. Offer assistance.
**Contemplation**
In this stage, landlords may be interested, but not ready to change. They may be afraid to take action, or they do not know how to go about doing it. Potential smoke-free housing activities for local coordinators to conduct in their communities with landlords in the contemplation stage were the following:
Be very useful when landlords call for help. Meet landlords to get them thinking about their process. Ask them to spell out pros and cons to smoke-free housing, and think about where they are on a scale of 1 to 10. Ask: what would move them to the next level? Share concrete stories about companies who have successfully gone smoke-free, with quotes about money saved and long-term benefits. Local stories would be good. Follow up periodically to offer assistance.
**Preparation**
In this stage, landlords would like to adopt a policy soon. They are talking about it within the company and talking to other landlords who have done it. They may be conducting a tenant survey or accessing available tools. Potential smoke-free housing activities for local coordinators to conduct in their communities with landlords in the preparation stage were the following:
Make sure you keep updated on tools available on websites such as smokefreehousinginfo.com and smokefreehousingnw.org. Provide sample lease language, sample tenant letters, and enforcement tools. Offer to help staff conduct resident meetings to discuss the no-smoking rule. Offer to help landlords conduct a tenant survey.
**Action**
In this stage, landlords are drafting language for leases, addendums, or house rules. They are informing tenants and staff about the rules and signing new leases with tenants. Potential smoke-free housing activities for local coordinators to conduct in their communities with landlords in the action stage were the following:
Share enforcement tools with landlords. Help landlords get local news story or other positive publicity. Be creative! Encourage landlords to put “smoke-free” in ads. Get good ideas and quotes to share.
**Maintenance**
In this stage, landlords are communicating and enforcing the rule with tenants, putting “smoke-free” in ads as an amenity, and getting positive feedback about reduced costs, more applicants, and positive stories. Potential smoke-free housing activities for local coordinators to conduct in their communities with landlords in the maintenance stage were the following:
Continue encouraging landlords to put “smoke-free” in ads. Follow up to see how things are going. Find and meet with local landlords who have adopted smoke-free policies. Get quotes and suggestions. See if they are willing to talk to others. Help landlords get positive publicity for successful implementation. Write stories for local landlord newsletters.
This figure was adapted with permission from material developed by Health In Sight LLC.

## Interpretation

This project featured collaboration between public health advocates, landlord and renter advocacy groups, and other community stakeholders who shared project resources and credit for successes. Listening to and respecting stakeholder views, especially those of private and public landlords, not only allowed their perspectives to surface but also built trust, cooperation, and a willingness to work through difficult issues together. Involving a wide range of partners from the very beginning, working with all sides, and seeking meaningful, timely input created enormous investment by key players, which was crucial in moving 2 large landlords through the stages of change into implementation of smoke-free policies and establishing smoke-free housing as a community norm.

The key to success in the private sector was highlighting the business case for no-smoking policies. High renter demand for smoke-free housing, the desire to reduce smoking-related costs (lower turnover and maintenance costs), and the fact that no-smoking policies are legal were powerful arguments with property managers. Health-related arguments were not a motivating factor for landlords. Essential to moving landlords to action were having our messages carried by a champion who represented a leading company and an influential trade association and presenting our messages through existing industry communications. They knew that the information was credible because it came from trusted, familiar sources.

This project demonstrated the importance of using data to drive policy change. Newly collected local data were used to counter property managers' overestimation of the prevalence of smoking among renters and to show that renters not only supported no-smoking policies but that they also viewed smoke-free as a desired amenity. The landlord focus groups taught public health staff the importance of making the business case for policy change, what educational tools were needed, and what concerns landlords had regarding the legality of no-smoking policies.

Having seasoned staff and sufficient resources were essential for the success of this project. The initiating agencies and organizations provided experienced tobacco control personnel and gave them enough time to get the job done. In addition, grant funding allowed essential data collection and provided resources for meetings, workshops, development and distribution of educational materials and tools, and small contracts for partners. This project and projects in other states have created educational tools that are available to others who wish to develop smoke-free multiunit housing campaigns, making start-up much less expensive. However, projects should make sure they have sufficient funding and a well-prepared staff to collect local data and to develop relationships within the housing sector that are key to motivating landlords to adopt no-smoking policies.

One limitation of this case study is that our campaign took place in Oregon, an environment where secondhand smoke exposure is less acceptable because of a strong smoke-free workplace law and effective state and local tobacco control programs. It is not clear that a smoke-free multiunit housing campaign, even one that employs the lessons learned from this case study, will be successful in an environment without strong antitobacco laws and broad social norms supporting smoke-free environments.

Our experience suggests that property managers are competitive with one another and that they want their standard of practice to be of high caliber and attractive to renters. Thus, to the extent that no-smoking policies in rental housing become more normative, more and more landlords will follow suit. In this regard, we are encouraged to see that a memo from the US Department of Housing and Urban Development "strongly encourages Public Housing Authorities (PHAs) to implement non-smoking policies in some or all of their public housing units" (17). We hope that this case study will assist in the movement to ensure that all people, including those living in multiunit housing, are protected from exposure to secondhand smoke.

## Appendix. Portland-Vancouver Metro Area Smokefree Housing Project Teams

**Leadership team**

American Lung Association in Oregon
Clackamas County Community Health
Clark County Public Health
Multnomah County Health Department
Washington County Department of Health and Human Services

**Advisory board members**

Fair Housing Council of Oregon
Housing Authority of Portland
Housing Connections
IRCO/Asian Family Center
Josiah Hill III Clinic
Kennedy Restoration
The Landlord Times/Professional Publishing, Inc.
LifeWorks Northwest
Metro Multifamily Housing Association
Oregon Human Development Corporation
Oregon Rental Housing Association
Oregon Smokefree Housing Project
Portland Development Commission
Portland Housing Bureau
Tobacco Free Coalition of Clark County
Tobacco Free Coalition of Oregon
Tualatin Valley Fire and Rescue
Vancouver Housing Authority
